# Normative Data for the Balance Error Scoring System in Adults

**DOI:** 10.1155/2013/846418

**Published:** 2013-03-18

**Authors:** Grant L. Iverson, Michael S. Koehle

**Affiliations:** ^1^Department of Physical Medicine and Rehabilitation, Harvard Medical School, Boston, MA, USA; ^2^Red Sox Foundation and Massachusetts General Hospital Home Base Program, Boston, MA, USA; ^3^Copeman Healthcare Centre, Vancouver, BC, Canada V6Z 2L4; ^4^School of Kinesiology and Division of Sport Medicine, University of British Columbia, 210-6081 University Boulevard, Vancouver, BC, Canada V6T 1Z1

## Abstract

*Background*. The balance error scoring system (BESS) is a brief, easily administered test of static balance. The purpose of this study is to develop normative data for this test. *Study Design*. Cross-sectional, descriptive, and cohort design. *Methods*. The sample was drawn from a population of clients taking part in a comprehensive preventive health screen at a multidisciplinary healthcare center. Community-dwelling adults aged 20–69 (*N* = 1, 236) were administered the BESS within the context of a fitness evaluation. They did not have significant medical, neurological, or lower extremity problems that might have an adverse effect on balance. * Results*. There was a significant positive correlation between BESS scores and age (*r* = .34). BESS performance was similar for participants between the ages of 20 and 49 and significantly declined between ages 50 and 69. Men performed slightly better than women on the BESS. Women who were overweight performed significantly more poorly on the test compared to women who were not overweight (*P* < .0001; Cohen's *d* = .62). The BESS normative data are stratified by age and sex. *Conclusions*. These normative data provide a frame of reference for interpreting BESS performance in adults who sustain traumatic brain injuries and adults with diverse neurological or vestibular problems.

## 1. Introduction

Problems with balance and postural stability can arise from injuries or diseases affecting the vestibular system and/or brain. Examples include stroke [1], Parkinson's disease [2], multiple sclerosis [3], traumatic brain injury [4–10], blast exposure in the military [11], and sport-related concussion [12]. Blunt trauma to the head can cause persistent problems with dizziness and balance via a variety of vestibular system problems such as labyrinthine concussion, rupture of the round window membrane, delayed endolymphatic hydrops, and benign paroxysmal positional vertigo due to canalolithiasis [13]. 

The balance error scoring system (BESS; [10, 14–16]) is a rapid, relatively easy-to-administer, and inexpensive measure of static balance and postural stability. A combination of three stances (narrow double leg stance, single leg stance, and tandem stance) and footing surfaces (firm surface/floor or medium density foam) is used for the test. Iverson et al. [17] presented preliminary normative data for the BESS derived from community-dwelling adults (*N* = 589) between the ages of 20 and 69. The purpose of this study is to expand the normative reference data for this test for adults and older adults.

## 2. Methods

### 2.1. Participants

Community-dwelling adults (*N* = 1, 236) were administered the BESS within the context of a comprehensive preventive health screen at a multidisciplinary healthcare center. This sample included the original 589 subjects from Iverson et al. [17]. The average age of the sample was 49.5 years (SD = 10.8; range = 20–69). There were 739 men (59.8%) and 497 (40.2%) women. The average body mass index (BMI) for men was 28.1 (SD = 4.1, range = 16.2–49.0) and for the women was 24.5 (SD = 4.5, range = 15.9–45.8). All medical charts were reviewed by a medical student and physician to exclude individuals with significant neurological, medical, or lower extremity conditions that could potentially have affected their balance. 

### 2.2. Measure

**Figure 1:**
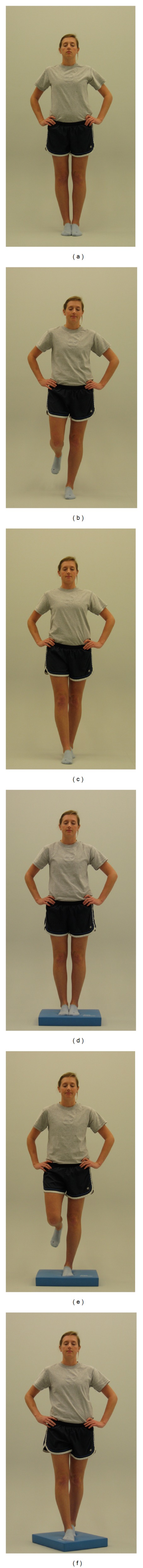
Balance error scoring system (BESS) performed on firm surface ((a)–(c)) and foam surface ((d)–(f)). Figure reprinted with permission from Davis et al. [24].

The Balance Error Scoring System (BESS; [10, 14–16]) uses a combination of three stances (narrow double leg stance, single leg stance, and tandem stance) and two footing surfaces (firm surface/floor or medium density foam). The first 3 stances, on a hard surface, comprise the test. Each stance is held, with hands on hips and eyes closed, for 20 seconds. “Error” points are given for specific behaviors, including opening eyes, lifting hands off hips, or stepping, stumbling, or falling. Therefore, a higher total score reflects worse performance on the test. All tests were scored by professional kinesiologists trained in the administration of the BESS and who perform the test daily as part of their practice. For reliability, these kinesiologists evaluated BESS performance from a video with an ICC = 0.88. BESS performance can vary, or be influenced by, a number of factors including the type of sport played [18], a history of ankle injuries and ankle instability [19], and exertion and fatigue [20, 21]. Uninjured athletes can have a subtle learning effect on the BESS when it is administered over brief retest intervals [22, 23]. The BESS is illustrated in Figure 1. 

## 3. Results

There was a small significant positive correlation between BESS scores and age (*r* = .34, *P* < .0001). Comparing the six age groups in Table 1, parametric and nonparametric analyses revealed an overall main effect for age (*F*(6, 1,235) = 30.6, *P* < .0001; Levene's test for homogeneity of variances, *P* < .0001; Kruskal-Wallis (6, 1,236) = 170.5, *P* < .0001; Independent-Samples Median Test (6, 1,236) = 128.8, *P* < .0001). BESS performance was similar for participants between the ages of 20 and 49 and significantly declined between ages 50 and 69. ANCOVA revealed slightly better BESS performance in men than in women (*F*(2, 1,235) = 5.4, *P* < .021, partial  eta  squared = .004). The effect size for this difference was very small, however. There was a nonsignificant trend for men who were overweight (BMI ≥ 30) to perform more poorly on the BESS (*t*(1, 736) = 1.92, *P* < .055, *d* = .16). Women who were overweight performed significantly more poorly on the test (*t*(1, 494) = 4.33, *P* < .0001; *d* = .62). The BESS normative data are provided in Table 1.

## 4. Discussion

Balance can be adversely affected by a wide range of vestibular or neurological illnesses, injuries, or conditions. Moreover, age-associated declines in computerized dynamic posturography are well documented in the literature [25, 26]. As seen in Table 1, balance and postural stability, as measured by the BESS, decline with age.

The BESS is a standardized, rapid, inexpensive, and screening test of postural stability that can be helpful for documenting deficits, monitoring recovery from injury, or tracking deterioration due to a neurological condition. It has been used in many studies with healthy athletes [18, 20–22, 27, 28], and as an outcome measure relating to ankle instability [19, 29, 30] or sport-related concussion [10, 14, 15]. A systematic review of the BESS, published in 2011, provides important information regarding its reliability and validity [31]. The normative data in the present study provide a frame of reference for interpreting BESS performance across the lifespan in healthy adults. These normative reference values allow the clinician to classify a person's balance across a broad range, from superior (top 10%) to very poor (bottom 2%).

The clinical use of the normative reference values in Table 1 is straightforward. A person's total score can be classified in certain ranges relative to the normative reference values. For example, a 52-year-old man with a score of 20 would be classified as having “below average” balance relative to his age (i.e., men and women combined) and relative to men his age. His score is in the lower 25% of the normative subjects. A 45-year-old overweight woman who sustained a moderate traumatic brain injury three months prior to testing obtained a score of 25. Compared to women of her age, her balance was “poor” (i.e., lower 10% of the normative subjects). Compared to a small sample of women who are overweight, her performance was below average (i.e., lower 25% of the normative sample). A 62-year-old man participating in a cardiac rehabilitation program following a myocardial infarction obtained a score of 7. Compared to men of his age, this score is in the “superior” range (i.e., upper 10% of the normative subjects). If a patient's balance is impaired, or the clinician decides that it might not be safe or necessary to test balance on the foam mat, then normative reference values for the Modified Balance Error Scoring System (M-BESS; first 3 stances on hard surface) can be used [32, 33]. At present, there are no published studies related to the test-retest reliability of the BESS in adults (with the exception of athletes) or older adults, so it is not possible to estimate test-retest measurement error. Therefore, there is no statistical approach available for interpreting change in BESS scores across time in rehabilitation settings. Rehabilitation professionals using the BESS must simply rely on clinical judgment for interpreting change scores until future reliable change data becomes available.

When using these normative data, clinicians should keep in mind that they were derived from a single laboratory in Vancouver, Canada. This was a reasonably healthy adult sample that underwent a fitness evaluation at a private healthcare center in Canada. The individuals in this sample have a higher than average socioeconomic status (SES), and a relatively small percentage of them were obese, so the results might not generalize across the full spectrum of SES or to adults who are overweight.

Computerized dynamic posturography may reveal abnormalities in postural responses to changing sensory conditions that are not detected by measures such as the BESS [34, 35]. Moreover, balance problems can be more apparent under conditions of physical exertion in some patients [36]. Therefore, in rehabilitation settings, it is important to monitor balance in the context of progressively increasing levels of physical exertion. 

## Figures and Tables

**Table 1:** Normative reference values for the BESS stratified by age.

Age	*N*	Mean	Median	SD	Superior	Above average	Broadly normal	Below average	Poor	Very poor
20–29	65	11.3	11.0	4.8	0–5	6-7	8–14	15–17	18–23	24+
30–39	173	11.5	11.0	5.5	0–4	5–7	8–15	16–18	19–26	27+
40–49	352	12.5	11.5	6.2	0–5	6–8	9–16	17–20	21–28	29+
50–54	224	14.2	12.0	7.5	0–6	7-8	9–18	19–24	25–33	34+
55–59	197	16.5	15.0	7.6	0–7	8–10	11–20	21–28	29–35	36+
60–64	148	18.0	16.5	7.8	0–8	9–12	13–22	23–28	29–40	41+
65-69	77	19.9	18.0	7.1	0–12	13–15	16–24	25–32	33–38	39+
Men										
20–29	26*	10.4	10.0	4.4	0–4	5-6	7–14	15	16–21	22+
30–39	97	11.5	11.0	5.5	0–4	5-6	7–15	16–18	19–26	27+
40–49	212	12.4	12.0	5.7	0–5	6-7	8–16	17–20	21–27	28+
50–54	142	13.6	12.0	6.9	0–6	7	8–17	18–23	24–28	29+
55–59	117	16.4	15.0	7.2	0–7	8–10	11–20	21–28	29–34	35+
60–64	89	17.2	16.0	7.1	0–8	9–11	12–21	22–27	28–35	36+
65–69	56	20.0	18.0	7.3	0–12	13-14	15–23	24–33	34–39	40+
Women										
20–29	39*	11.9	11.0	5.1	0–5	6-7	8–14	15–19	20–25	26+
30–39	76	11.4	10.5	5.6	0–4	5-6	7–15	16–19	20–27	28+
40–49	140	12.7	11.0	6.9	0–5	6-7	8–15	16–20	21–29	30+
50–54	82	15.1	13.0	8.2	0–7	8-9	10–20	21–24	25–35	36+
55–59	80	16.7	15.0	8.2	0–8	9-10	11–21	22–28	29–39	40+
60–64	59	19.3	17.0	8.8	0–9	10–12	13–22	23–31	32–43	44+
65–69	21*	19.9	18.0	6.6	0–13	14	15–24	25–27	28–38	39+
Women: BMI ≥ 30										
20–49	27*	17.3	16.0	6.5	0–8	9–12	13–22	23–27	28–33	34+
50–64	32*	21.6	20.0	8.4	0–11	12–14	15–27	28–32	33–41	42+

*Unusually small sample sizes limit the usefulness of these normative reference values. The maximum score for each trial was truncated at 10 points. BMI: body mass index. Body mass had a greater effect on balance performance in women than in men. Superior scores occur in fewer than 10% of the sample. Above average scores occur in approximately 15%, broadly normal scores occur in approximately 50%, below average scores occur in approximately 15%, poor scores occur in approximately 8%, and very poor scores occur in fewer than 2%. These classification ranges correspond to the following percentile ranks: Very poor < 2nd percentile; poor = 2nd–9th percentile; below average = 10th–24th percentile; broadly normal = 25th–75th percentile; above average = 76th–90th percentile; superior > 90th percentile.
